# Breast Cancer and Tumor Microenvironment: The Crucial Role of Immune Cells

**DOI:** 10.3390/curroncol32030143

**Published:** 2025-02-28

**Authors:** Tânia Moura, Paula Laranjeira, Olga Caramelo, Ana M. Gil, Artur Paiva

**Affiliations:** 1 Flow Cytometry Unit, Department of Clinical Pathology, Hospitais da Universidade de Coimbra, Unidade Local de Saúde de Coimbra, 3000-076 Coimbra, Portugal; tania.moura@ua.pt (T.M.); 1979paula@gmail.com (P.L.); 2 Department of Chemistry, University of Aveiro, 3810-193 Aveiro, Portugal; agil@ua.pt; 3 Group of Environmental Genetics of Oncobiology (CIMAGO), Coimbra Institute for Clinical and Biomedical Research (iCBR), Faculty of Medicine (FMUC), University of Coimbra, 3000-548 Coimbra, Portugal; 4 Center for Innovative Biomedicine and Biotechnology (CIBB), University of Coimbra, 3000-504 Coimbra, Portugal; 5 Clinical Academic Center of Coimbra (CACC), 3000-061 Coimbra, Portugal; 6 Center of Neurosciences and Cell (CNC), University of Coimbra, 3000-504 Coimbra, Portugal; 7 Gynecology Department, Hospitais da Universidade de Coimbra, Unidade Local de Saúde de Coimbra, 3000-075 Coimbra, Portugal; olgalgcaramelo@gmail.com; 8 CICECO—Aveiro Institute of Materials, Department of Chemistry, University of Aveiro, 3810-193 Aveiro, Portugal; 9 Ciências Biomédicas Laboratoriais, Instituto Politécnico de Coimbra, ESTESC—Coimbra Health School, 3046-854 Coimbra, Portugal

**Keywords:** breast cancer, tumor microenvironment, immune system, immune cells, immune checkpoints inhibitors

## Abstract

Breast cancer is the most common type of cancer in women and the second leading cause of death by cancer. Despite recent advances, the mortality rate remains high, underlining the need to develop new therapeutic approaches. The complex interaction between cancer cells and the tumor microenvironment (TME) is crucial in determining tumor progression, therapy response, and patient prognosis. Understanding the role of immune cells in carcinogenesis and tumor progression can help improve targeted therapeutic options, increasing the likelihood of a favorable prognosis. Therefore, this review aims to critically analyze the complex interaction between tumor cells and immune cells, emphasizing the clinical and therapeutic implications. Additionally, we explore advances in immunotherapies, with a focus on immune checkpoint inhibitors.

## 1. Introduction

The tumor microenvironment (TME) refers to the highly complex, dynamic, and multifaceted ecosystem surrounding tumor cells, encompassing a diverse array of various cell types, extracellular matrix components, signaling molecules, immune cells, and blood vessels [1]. In breast cancer, the TME plays a critical role in tumor progression, metastasis, and resistance to therapies. It is composed of immune cells, fibroblasts, endothelial cells, and the extracellular matrix, which interact with tumor cells through direct contact and soluble factors [2]. These interactions facilitate tumor growth, immune evasion, and the establishment of a supportive environment for cancer cell survival and proliferation [3]. The TME is not merely a passive bystander but an active participant in cancer biology. Its influence extends to treatment outcomes, as it can modulate the response of cancer cells to conventional therapies, including chemotherapy, targeted treatments, and immunotherapy [4,5]. Understanding the components and dynamics of the TME is therefore crucial for developing more effective therapeutic strategies that not only target the tumor cells but also the microenvironment that supports them.

## 2. Breast Cancer

Breast cancer, one of the main causes of morbidity and mortality in females worldwide, represents a serious public health problem [6,7]. Statistics indicate that in 2022, approximately 2.3 million women were diagnosed with breast cancer worldwide, with over 660 thousand resulting in death, representing a considerable proportion of the overall cancer cases [7,8,9]. Despite recent advances in diagnostic and treatment strategies, mortality rates remain high, which highlights the demand for new therapeutic strategies [7,10]. Concomitantly, epidemiological studies have associated a higher probability of developing breast cancer with various risk factors such as genetic mutations, radiation exposure, age of menarche, and unhealthy lifestyle habits [11,12]. The causes behind this disease are still not fully understood, making it harder to develop prevention strategies and new therapy approaches [13,14].

Breast cancer is recognized as a heterogeneous disease, comprising various tumor types with distinct cellular and molecular features, leading to differing prognoses and therapeutic interventions [15,16]. The clinical categorization of breast cancer is based on the expression status of specific receptors, including the estrogen receptor (ER), progesterone receptor (PgR), human epidermal growth factor receptor 2 (HER2), and the Ki-67 proliferation marker protein [13,17,18]. This classification results in four main subtypes which can be grouped into surrogate intrinsic subtypes, namely luminal A-like, luminal B-like, HER2 positive, and triple negative. Luminal A-like tumors are typically of a low grade, strongly ER positive/PgR positive, HER2 negative, and are characterized by a low proliferation. Luminal B-like tumors are hormone receptor (HR) positive but may have variable degrees of ER/PgR expression, being characterized by a higher grade and higher proliferation than luminal A tumors [14,19]. Luminal A breast cancer typically carries a more favorable prognosis compared to other subtypes, while triple negative breast cancer is known for its aggressive nature and resistance to conventional treatments [17]. Each subtype warrants tailored first-line therapies. Luminal tumors often respond well to endocrine therapy, HER2-positive tumors benefit from anti-HER2 therapy, while the most effective approach for managing triple negative breast cancers continues to be chemotherapy [14,17]. In this context, recent advances in targeted therapies, such as antibody–drug conjugates, selective estrogen receptor degraders, and nanotechnology-based drug delivery systems, have emerged as promising approaches to overcome resistance mechanisms and improve treatment efficacy in hormone receptor-positive, HER2-negative breast cancer. Additionally, the repurposing of approved drugs has gained attention as a cost-effective and time-efficient strategy to expand treatment options [20]. Immunotherapy has emerged as a promising targeted therapy in breast cancer treatment, despite the historically low immunogenicity of these tumor cells compared to other tumors [21]. The use of immunotherapy-based strategies has revolutionized treatment paradigms. As a result, in the pursuit of more effective treatments, researchers have increasingly focused on the role of the tumor microenvironment (TME) in tumor progression and in shaping immune response [19,21].

## 3. Tumor Microenvironment

The TME represents a complex and heterogeneous ecosystem, comprised of various stromal cells, such as fibroblasts, mesenchymal cells, adipocytes, and immune cells. This intricate network may create an environment conducive to the growth and proliferation of cancer cells [22,23]. Interactions among these cells within the TME involve the secretion of cytokines and growth factors, which significantly influence the processes of carcinogenesis, tumor progression, and the response to treatment in breast cancer [24].

Throughout the progression of breast cancer, the TME undergoes dynamic changes that can impact the disease course and treatment outcomes. Emerging research suggests that cells within the TME can serve as biomarkers for monitoring treatment response, reflecting the immune status of tumor cells and influencing tumor development [4,5]. Additionally, manipulating the composition of TME cells presents an opportunity to enhance the immune response against the tumor. However, despite current progress, the crosstalk between cancer cells and TME components remains incompletely understood [25].

In particular, a comprehensive understanding of the intricate interaction interplay between breast cancer cells and immune cells within the TME holds significant promise for the development of increasingly targeted and effective therapies against various subtypes of breast cancer. Thus, this review aims to elucidate the current advances in this complex interaction, emphasizing the clinical and therapeutic implications of immune cells. By elucidating this interaction, researchers can pave the way for the development of innovative treatment strategies tailored to different breast cancer subtypes.

## 4. Decoding the Multifaceted Role of Immune Cells in the Breast Cancer Tumor Microenvironment

The impact of immune cells on the development and survival of breast cancer tumors varies depending on the tumor subtype and the composition of inflammatory cells within the TME. Triple negative breast cancer is notably immunogenic, standing out among the remaining subtypes [26]. Malignant breast cells interact with various cells of the immune system, influencing tumor proliferation, development, progression, and the efficacy of therapeutic interventions [27,28].

**Figure 1:**
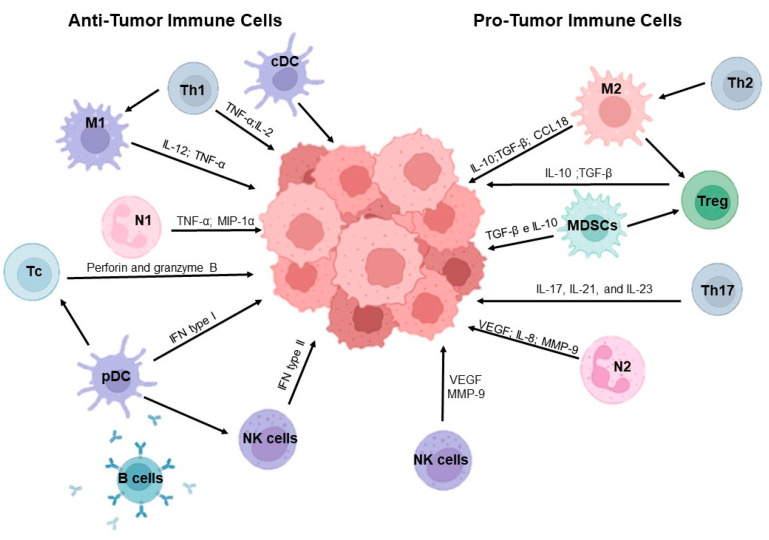
The interaction between various cells that make up the immune system and the tumor microenvironment. cDC—conventional dendritic cells; IL—interleukin; M1—M1 macrophages; M2—M2 macrophages; MDSCs—myeloid-derived suppressor cells, MIP-1-alpha—macrophages inflammatory protein-1-α; MMP-9—matrix metalloproteinases-9; N1—N1 neutrophils; N2—N2 neutrophils; NK—natural killer cells; pDC—plasmacytoid dendritic cells; Tc—cytotoxic T cells; Th1—H helper cells 1; TNF-α—tumor necrosis factor alpha; Treg—regulatory T cells; VEGF—vascular endothelial growth factor. Created with BioRender.com.

Immune cells within the TME exert a dual influence, with roles in both the inhibition of tumor progression and the promotion of immunosuppression and tumor cell growth (Figure 1) [22]. Within the diverse populations of immune cells, macrophages, neutrophils, natural killer (NK) cells, dendritic cells, myeloid-derived suppressor cells (MDSCs), innate lymphoid cells (ILCs), and lymphocytes are abundant, each contributing uniquely to tumor dynamics and responses to therapy.

### 4.1. Innate Immunity: Macrophages, NK Cells, Neutrophils, MDSCs, Dendritic Cells, and ILCs

#### 4.1.1. Tumor-Associated Macrophages

Tumor-associated macrophages (TAMs) are pivotal components on the TME in breast cancer, exerting a profound influence on disease progression. These cells constitute a substantial portion on the TME and play diverse roles in carcinogenesis, immunosuppression, and chemotherapy resistance, thereby impacting the metastatic potential of the tumor and the clinical course and outcome of the patients [29]. Chemokine signals are known to attract TAMs to the TME, where they secrete pro-tumoral factors, such as the vascular endothelial growth factor (VEGF), thus fostering angiogenesis and cancer cells survival [30].

TAMs exhibit a remarkable plasticity and can adopt either classically activated TAMs (M1) or alternatively activated (M2) phenotypes. While M1 TAMs possess anti-tumor properties, M2 TAMs promote immunosuppression and angiogenesis [31]. In breast cancer TME, TAMs predominantly assume the M2 phenotype, contributing to cancer cell survival and dissemination through the secretion of cytokines (namely, interleukin (IL)-10, transforming growth factor (TGF)-β, and CCL18. They also induce regulatory T (Treg) cells upon interaction with CD4+ T cells [30,32].

The relationship between TAM density, activity, and breast cancer prognosis is notable, albeit with nuances based on clinicopathological characteristics [29]. In relation to M1 macrophages, these cells have been demonstrated to exhibit some anti-tumor activity, inhibiting the invasion and proliferation of breast cancer cells in vitro [33]. Previous studies have consistently linked the presence of M2 TAMs with an unfavorable prognosis, with increased M2 density correlating significantly with poorer clinical outcomes and reduced treatment efficacy [28,29,34,35,36]. Meta-analyses have further demonstrated a higher proportion of M2 macrophages in breast tumors with high histological grades and a higher Ki-67 proliferating index [37]. In triple negative breast cancer, TAMs are particularly associated with metastasis development, exhibiting significantly increased densities compared to other subtypes [34,38]. Moreover, recent evidence suggests a negative correlation between M2 macrophages and the response to neoadjuvant chemotherapy [28].

Macrophages are also capable of differentiating into regulatory macrophages (Mreg), hemophagocytic macrophages (Mha), oxidative macrophages (Mox), M3, M4, and M17. Mreg produces IFN-γ, suppresses T lymphocyte proliferation, and activate regulatory T cells [39]. Emerging research has identified M3 macrophages as a distinct population still under characterization. Studies indicate that this cell population plays distinct roles from those known for M1 and M2 macrophages. Indeed, M3 macrophages have been described as having both immunomodulatory and pro-inflammatory characteristics [40]. The specific function of M3 macrophages in breast cancer is still under investigation, although these cells have been reported to exhibit anti-tumor activity in Ehrlich ascites [41]. Similarly to M3 macrophages, M4 macrophages represent a recently identified group of cells. M4 macrophages are differentiated in the presence of colony-stimulating factor (CSF) and CXCL4 [39]. M4 macrophages are atherogenic, as they produce pro-inflammatory cytokines such as IL-6 and TNF-α. In addition, M4 macrophages have been shown to be weakly phagocytic and are distinguished by high levels of MMP-7 [42,43]. However, the exact functions of M4 macrophages remain unclear. Further research is necessary to fully understand the contributions of both M3 and M4 macrophages in breast cancer and their potential as therapeutic targets.

Recent advances in TAMs research have spurred the development of therapies specifically targeted to macrophages, some of these presently undergoing clinical trials for breast cancer [44]. These interventions encompass strategies such as curbing macrophage recruitment, reshaping TAMs towards an anti-tumoral phenotype, and bolstering macrophage-mediated phagocytosis or the induction of tumor cell death [30].

#### 4.1.2. Natural Killer Cells

Natural killer (NK) cells exhibit unique immunological properties, blending characteristics of both innate and adaptive immunity [45]. Regardless of their classification, NK cells are identified by their expression of the surface protein NK1.1, constituting 5% to 10% of lymphocytes in human peripheral blood [46]. In addition to directly inducing tumor cell death, NK cells also bolster the demise of malignant cells by releasing cytokines and chemokines that activate and recruit other components of the immune system [45,47].

Numerous studies have correlated the presence of NK cells in the TME with a favorable prognosis in breast cancer patients [48,49]. However, despite this positive association, evidence suggests that NK cells can undergo alterations within the tumor milieu, including the upregulation of inhibitory receptors [50] and the expression of VEGF and matrix metalloproteinase 9 (MMP9) [51], which may compromise their anti-tumor efficacy. Furthermore, a higher infiltration of NK cells has been demonstrated in tissue samples from patients with triple negative breast cancer compared to other breast cancer subtypes [52].

The survival, maturation, and activation of NK cells are influenced by the secretion of cytokines originating from tumor or stromal cells [53,54]. Among these cytokines, interferon (IFN) type II stands out, playing a pivotal role in regulating both adaptive and innate immune responses. Type II interferon orchestrates a cascade of immune anti-tumor effects, including the modulation of effector cells and the induction of T cell memory. In solid tumors, such as breast cancer, the diminished responsiveness of NK cells to interferon significantly compromises their function [55]. This is particularly relevant in subtypes such as luminal A, where NK cells activity may be more robust, potentially, influencing treatment outcomes [56].

Several studies have shown that breast cancer patients have NK cells with a reduced cytotoxic activity and a CD56dim and perforin high phenotype [57]. Analyses indicate that although the total proportion of NK cells is similar in malignant and normal breast tissues, it is lower in normal tissue [47]. In contrast, the proportion of CD56bright NK cells is higher in malignant breast tissues, and these cells show a higher expression of activation markers such as NKp44 [58]. Consequently, in breast cancer, the anti-tumor and cytotoxic activity of NK cells seem to be reduced. Additionally, these cells exhibit an inhibitory phenotype, including KIR-3DL1 and KIR-2DL3, and have a lower expression of activation receptors, such as NCRs, NKG2D, NKp30, DNAM-1, and CD16. These phenotypic changes, therefore, correlate with the decreased function of NK cells, contributing to tumor progression [47,59].

In the case of luminal A breast cancer, a notable trend toward a higher frequency of NK cells was observed when compared to the luminal B. This finding suggests the existence of potential differences in the immune microenvironment between these two subtypes, which may reflect distinct patterns of immune cell infiltration and regulation [56]. Given the known ability of NK cells to secrete cytokines and chemokines that activate other immune components, this trend might have implications for the broader immune response and tumor dynamics in luminal A tumors.

Hence, functional alterations and interactions within the TME significantly contribute to the complexity of immune regulation and have a profound impact on clinical outcomes, indicating tumor progression, treatment response, and overall patient survival [54]. Given these complexities, further research is warranted to fully understand the multifaceted role of NK cells within the TME, as well as their potential therapeutic implications in improving patient’s outcomes.

#### 4.1.3. Tumor-Associated Neutrophils

Neutrophils are the most abundant leukocytes in human circulation, constituting a significant portion of the TME in various types of cancers [60]. Traditionally viewed as frontline defenders against infections, the biological roles of neutrophils have often been underestimated due to their short lifespan, typically around a few hours. However, recent research has shed light on the classification of tumor-associated neutrophils into anti-tumor (N1) and pro-tumor (N2) phenotypes [60,61,62].

N1 neutrophils exhibit an enhanced phagocytic capacity, efficient migration, and cytotoxicity against tumor cells. These cells release tumor necrosis factor (TNF)-α and CC motif chemokine ligand 3 (CCL3, formerly known as macrophage inflammatory protein-1α, MIP-1α), exerting suppressive effects on the tumor growth [63]. In contrast, N2 neutrophils suppress T cell activity, display a reduced cytotoxicity against tumor cells, and secrete high levels of pro-tumoral factors, like VEGF, IL-8, and MMP-9, thus promoting angiogenesis, tumor growth, and metastasis [64].

Despite playing fundamental roles in carcinogenesis and tumor progression, limited knowledge on the specific functions of neutrophils in breast cancer remains [63]. Pro-tumoral neutrophils have been implicated in various stages of breast cancer development, including tumor initiation, metastasis, and immunosuppression, and have been identified in the TME of multiple cancer types, including breast cancer [65]. However, the precise role of neutrophils depends on various factors such as tumor stage and the presence of other immune cells.

Studies have shown that TGF-β, present in high concentrations in the primary TME, promotes the accumulation of pro-tumoral neutrophils polarized towards a N2 phenotype within the tumor. This shift towards an N2 phenotype contributes to a more immunosuppressive TME, facilitating tumor growth and metastasis. Conversely, blocking TGF-β, along with type I interferons, can shift the balance towards a more anti-tumoral phenotype [61,66]. In patients with triple negative breast cancer, a higher proportion of neutrophils is found, primarily due to the presence of certain cytokines like TGF-β, which stimulate the production of neutrophils in bone marrow and their migration to the TME [67].

Several studies have established a correlation between tumor-associated neutrophils and favorable prognostic parameters, such as the overexpression of PgR, a low proliferative index, and a low histological grade, as well as longer survival [68]. Conversely, Kakumoto and collaborators demonstrated that a high frequency of tumor-infiltrating neutrophils is associated with more advanced histological grades, larger tumor size, and the molecular subtypes of triple negative and HER2+ breast cancer [69]. However, it is important to note that most studies do not evaluate the subtype of neutrophils infiltrating the tumor.

Furthermore, the ability of neutrophils to interact with other immune cells in the TME suggests their potential as therapeutic targets. Modulating neutrophil polarization or enhancing the cytotoxic potential of N1 neutrophils could potentially improve immune-based therapies in breast cancer, particularly in the context of immune checkpoint inhibitors and targeted therapies.

#### 4.1.4. Myeloid-Derived Suppressor Cells

Another type of cellular subpopulation found in the TME is the myeloid-derived suppressor cells (MDSCs). These cells are immature and dysfunctional components of the innate immune system that can suppress T cells and NK cells, contributing to cancer cell progression and metastasis [70]. MDSCs mediate immunosuppression through various mechanisms, such as the induction of oxidative stress via the secretion of reactive oxygen species (ROS) and the release of factors like VEGF, MMPs, TGF-β, and IL-10, influencing angiogenesis [71].

These cells exhibit a complex phenotype that is not limited to a single defined population, making identification challenging. MDSCs can be classified into two main subsets: monocytic MDSCs (M-MDSCs) and polymorphonuclear MDSCs (PMN-MDSCs, also known as granulocytic MDSCs) [72]. Cancer cells recruit MDSCs to the TME through various cytokines, such as CCL1, CCL2, CCL5, and CXCL5 [73,74]. In breast cancer, an estrogen-rich environment contributes to this recruitment [75].

Clinically, an increase in MDSCs correlates with the stage of cancer and tumor burden [76]. Specifically in breast cancer, elevated levels of MDSCs, especially M-MDSCs, have been associated with metastatic status [77,78]. Studies reported that MDSCs are more abundant in samples from patients with triple negative breast cancer compared to those with non-triple negative types [79]. Furthermore, it has been proposed that PMN-MDSCs constitute the largest fraction in many types of cancer, becoming predominant in triple negative breast cancer [77,80]. However, the composition of the immune infiltrate varies across studies and there are controversies regarding the role of M-MDSCs. Some studies reported a higher number of these cells in blood [77], while others did not observe this expansion either in blood or in the TME [81]. Overall, the presence of MDSCs has been correlated with an advanced disease status and poorer overall survival [76,80]. However, further studies are needed to better understand the composition of the immune infiltrate in breast cancer and its different molecular subtypes.

The contribution of MDSCs to the immune suppression in the TME is particularly significant in breast cancer, as these cells can interfere with T cell activation, suppress NK cell function, and promote an environment that facilitates tumor progression. However, further studies are needed to better understand the composition of the immune infiltrate in breast cancer and its different molecular subtypes.

#### 4.1.5. Dendritic Cells

Dendritic cells (DCs) represent a heterogeneous group of innate immune cells crucial for activating and modulating the adaptive immune response [82]. These cells can be categorized into various subsets, namely conventional DCs (cDCs)—comprising cDC1 and cDC2—and plasmacytoid DCs (pDCs) [82,83]. cDCs are known as potent antigen-presenting cells and strongly induce T cell immune responses [82,84]. Conversely, pDCs are major producers of type I interferon and have been associated with anti-tumor activity induction through the direct activation of other immune cells, namely CD8 T cells and NK cells. However, pDCs can also contribute to tumor growth through the expression of immunosuppressive molecules like PD-L1 and ICOSL or the promotion of Treg expansion [83,85].

Notably, an upsurge in pDCs has been noted in the peripheral blood of breast cancer patients, particularly in HER2+, indicating distinctions among molecular subtypes. Nevertheless, the prognostic significance of these cells remains elusive, largely owing to the heterogeneous composition of breast cancer and staging [86]. In breast tumors, an infiltration of pDCs has been observed, wherein an increasing frequency of tumor-infiltrating pDCs was correlated with poorer prognosis [87]. However, recent studies have presented conflicting findings regarding the role of DCs in breast cancer. Some studies have reported associations between high levels of cDCs or pDC in triple negative tumors and more active immune microenvironments [88].

Understanding the distinct roles of DCs in different subtypes of breast cancer may become a valuable tool for patient stratification, biomarker development, and the design of targeted therapies.

#### 4.1.6. Innate Lymphoid Cells

Innate lymphoid cells (ILCs) represent an important class of cells in the innate immune system, divided into ILC1, ILC2, and ILC3. This classification is based on the expression of specific cytokines and transcription factors, with each subtype playing distinct roles in the context of immune response and modulation of the TME [89].

ILC1 expresses the transcription factor T-bet and produces type I cytokines, such as IFN- γ and TNF-α [89,90,91]. These cells are known for their potent cytotoxic activity against tumor cells, like NK cells. Studies have shown that in animal models of pre-cancerous breast lesions, ILC1 expands and exhibits cytotoxic activity against neoplastic cells, limiting tumor growth [92].

On the other hand, ILC2 produces effector cytokines such as IL-5, IL-9, and IL-13, which generally have a pro-tumor effect [90,91]. For example, IL-33 promotes tumor development in some models, and its serum levels increase in mammary carcinomas [93]. Additionally, IL-13 induces tumor progression and enhances macrophages polarization to the M2 phenotype, favoring the immune escape of tumor cells [94]. Notably, malignant breast tissue presents an enrichment of ILC2 compared to benign tissue [95].

ILC3 expresses the transcription factor RORγt and secretes IL-17 and IL-22 [90,91]. These cells play a dual role in carcinogenesis. IL-17 produced by ILC3 is associated with angiogenesis [96]. Conversely, IL-22 has been associated with a reduced breast tumor growth, indicating a possible antagonistic effect [97]. Studies have shown that in the TME of breast cancer, the presence of an increased number of ILC3 correlates with a higher likelihood of lymph node metastasis [96].

Recently, the anti-tumor role of ILCs has been documented in various types of tumors, including breast cancer [92]. However, the complexity of ILC interactions with the TME remains under investigation. A deep understanding of these mechanisms is essential for developing new therapeutic strategies that can exploit the anti-tumor functions of these cells.

### 4.2. Adaptative Immunity: T Lymphocytes and B Lymphocytes

The presence of tumor-infiltrating lymphocytes (TILs) in breast cancer signifies a host immune response against tumor cells [98]. These TILs serve as markers of prognosis and treatment response and are prominent components within the TME [99]. However, the prevalence of TILs varies significantly among breast cancer subtypes, with triple negative and HER2+ tumors typically demonstrating a higher lymphocytic infiltration compared to luminal subtypes [98,100,101].

While a positive correlation exists between TIL infiltration and prognosis in triple negative tumors (increased levels of TILs are associated with a better prognosis), the clinical relevance of TILs in luminal cancer is more limited [102]. Additionally, the quantity of TILs tends to decrease as the disease progresses. To enhance the understanding of their clinical relevance, the International TILs Working Group has advocated for standardization in TIL assessment [103].

A joint analysis of 3771 patients with a high number of TILs also shows an increased likelihood of response to neoadjuvant chemotherapy across all molecular subtypes stratified with neoadjuvant therapy [26].

TILs primarily comprise CD8+ cytotoxic T cells, CD4+ helper T cells, and regulatory T cells (Tregs). Interpreting the clinical relevance of TIL assessment in breast carcinoma is complex due to the high heterogeneity of this cancer type. Nonetheless, given the significance of TILs, efforts to comprehend the role of different subpopulations, particularly CD8, CD4, and Tregs, have been intensified [102].

#### 4.2.1. Helper T Lymphocytes

CD4+ T cells, generally known as helper T cells (Th), are versatile and multifunctional cells capable of differentiating into various functional subtypes [46]. They play a central role in anti-tumor activity by eliminating tumors through the release of IFN-γ and TNF-α [104].

Helper T lymphocytes encompass various subsets, whose development and functions are extensively described elsewhere [46,105]. However, the precise contribution of these subtypes to anti-tumor immunity remains incompletely understood. These cells produce elevated levels of IFN-γ and numerous other cytokines (namely TGF-β, TNF-α, and IL-2), thereby activating the M1 tumor-associated macrophages and cytotoxic T cells [106]. Consequently, the combined action of cytokines secreted by Th1 cells induces senescence in cancer cells [104]. Conversely, the role of Th2 cells is nuanced. Although traditionally associated with highly proliferative characteristics through the secretion of IL-4, IL-6, and IL-10, which activate M2 macrophages [107,108], a recent study suggests that Th2 cells can directly block mammary carcinogenesis by inducing a terminal differentiation of breast cancer cells [109]. Th9 and Th17 lymphocytes, while identified in various cancers, exhibit controversial roles, with reports indicating both pro-tumor and anti-tumor activities [110,111]. Th17 cells secrete pro-inflammatory cytokines, like IL-17, IL-21, and IL-23, regulating immune cell recruitment and stimulating inflammatory mediators and metalloproteinases, thereby influencing breast cancer prognosis. High levels of IL-17 producing cells are directly correlated with a high histological grade and the triple negative molecular subtype [110]. However, the exact nature of Th17 cells in breast cancer patients is still poorly understood. Th17 cells synergize with IFN-γ and IL-17 to induce the production of CXCL9 and CXCL10 [112]. It is important to note that these generalizations should be approached with caution given the plasticity of T cell subsets, as it has also been demonstrated that Th17 cells may participate in tumor eradication [111].

Th22 cells represent a newly identified subset of Th lymphocytes, and our understanding of their role in breast cancer remains limited. However, emerging evidence suggests their association with tumor progression across various cancer types, including gastric and ovarian cancers [113,114]. This association is attributed to the secretion of IL-22 by Th22 cells, which appears to inhibit apoptosis and stimulate the proliferation of tumor cells [115]. Furthermore, follicular helper T (Tfh) cells, characterized by CD279 (or programmed death 1, PD-1) and CD185 (or CXCR5) expression, play pivotal roles in humoral immunity regulation [116]. Recent research underscores their emerging significance in anti-tumor immunity, particularly in breast cancer [117].

In our previous work, we observed a significant increase in CD4+ T cells and Th1 cells in luminal A breast cancer compared to luminal B. Additionally, a slight positive correlation between Ki-67, a marker of cellular proliferation, and Th1 cells was identified within the luminal A subtype, suggesting that Th1-mediated immune responses might influence the proliferative capacity of these tumors [56]. This result contrasts with luminal B tumors, where such a correlation was not evident, potentially reflecting the distinct tumor biology and immune microenvironment between these subtypes. These findings highlight the relevance of Th1 cells in the tumor microenvironment, given their known role in the secretion of IFN-γ and TNF-α, which contribute to anti-tumor immunity [104]. This underscores the importance of considering immune landscape variations across molecular subtypes of breast cancer to better understand disease progression and therapeutic responses.

#### 4.2.2. Regulatory T Lymphocytes

Regulatory T (Treg) cells are specialized CD4+ T lymphocytes that play a crucial role in modulating the immune system to prevent autoimmune responses [46]. They are characterized by the high expression of CD25, low expression of CD127, and the presence of the transcription factor FoxP3 [118]. Tregs suppress the activity of other immune cells, inducing immunosuppression within the TME through various pathways, mediated by membrane-bound immunosuppressive molecules and by the secretion of soluble immunosuppressive cytokines, like IL-10 and TGF-β, thereby promoting tumor growth and metastasis [78].

Several studies have linked the presence of Tregs to an unfavorable prognosis and inadequate treatment response in breast cancer patients [119,120,121]. For instance, Papaioannou et al. identified elevated Treg levels in the TME of metastatic breast cancer patients compared to those without metastasis [122]. However, in early mammary carcinogenesis stages, Tregs may play a protective role, hindering the transition from pre-invasive to invasive breast cancer [123]. Notwithstanding, Treg infiltration is associated with a worse prognosis, especially in luminal tumor subtypes, where the simultaneous absence of cytotoxic T lymphocytes exacerbates the situation [124].

The heterogeneity of Treg cells provides various mechanisms of immune modulation contributing to the formation of an immunosuppressive TME [125]. Therefore, precise Treg characterization is crucial. Additionally, alongside Tregs, follicular regulatory T (Tfr) cells have emerged as a relevant cell class in regulating humoral immunity. Tfr cells suppress the function of follicular helper T (Tfh) cells and B cells, playing a crucial role in adaptive immune response regulation [126]. Patients with breast cancer, especially those with early-stage triple negative subtypes, exhibit increased Tfr levels in peripheral blood [100]. Both Tregs and Tfr cells are found in higher quantities in the breast tissue of cancer patients, compared to healthy individuals, and their presence is associated with lower survival rates [127]. Notably, Treg cell levels in tumor increase as the tumor progresses, further highlighting their role in breast cancer progression [128].

#### 4.2.3. Cytotoxic T Lymphocytes

Recent studies have underscored the importance of CD8+ cytotoxic T cells (Tc) in the TME of breast cancer. CD8+ T cells, as primary effectors of the adaptive immune system, play a crucial role in recognizing and eliminating tumor cells [31]. Tc cells are essential markers of the anti-tumor immune response, inducing apoptosis of target cells via cytotoxin release (such as perforin and granzyme B) and producing pro-inflammatory cytokines, like IFN-γ and TNF-α. Moreover, Tc cells can differentiate into various subtypes, each with distinct cytokine expression profiles and cytotoxic capabilities, present in the tumor in varying proportions [31,129].

Studies have shown a positive correlation between high levels of Tc cell infiltration and favorable clinical outcomes in breast cancer [130,131,132]. The presence of these cells correlates with a decreased density of ER and PgR receptors and an increased HER2 expression [132]. Elevated Tc cell levels in tumor tissue consistently relate to positive clinical outcomes across different breast cancer subtypes [102,133].

Furthermore, as Tc cells can suppress tumor angiogenesis through IFN-γ secretion, they can positively impact disease progression [104]. Thus, the presence of these cells may serve not only as an indicator of immune response efficacy against cancer but also have implications for prognosis and treatment response.

#### 4.2.4. γδ T Lymphocytes

Gamma delta (γδ) T cells represent a unique class of T lymphocytes with a crucial role in the anti-tumor immune response. Their T cell receptor (TCR) consists of a γ chain and a δ chain, endowing them with remarkable cytotoxic and pro-inflammatory properties that enable them to combat tumor cells [134,135].

The γδ T lymphocytes exert their cytotoxic activity against tumor cells through multiple mechanisms, including the secretion of perforin and granzyme B, as well as the Fas/FasL pathway. However, contrasting data have been demonstrated in recent publications regarding the roles played by these cells in tumor progression or suppression [136].

Based on differences in the γ and δ chains, γδ T cells can be divided into several subgroups: Vδ1 T cells, Vδ2 T cells (also referred to as Vγ9Vδ2), and Vδ3 T cells [137]. These subgroups have been found in solid tumors such as colon, lung, and breast cancer, where they participate in the immune response against tumor cells [138,139,140].

The role of these subgroups in the tumor microenvironment of breast cancers remains to be clarified. Contrary to the findings of Wu et al. [141], who identified Vδ1 cells as the main γδ T cell population in healthy breast tissue, it has been reported that Vδ2 T cells are the predominant subpopulation in triple negative breast cancer [142]. Additionally, the function of these cells in the context of breast cancer is not fully understood. Some studies suggest that Vδ1 T cells participate in immunogenic tolerance and can promote breast cancer progression by suppressing T cells [143]. On the other hand, Vδ2 T cells play important roles in anti-tumor immunity. In breast cancer, these cells have demonstrated cytotoxic functions against tumor cells and attenuated angiogenic signaling processes [144].

The Vδ3 T cells are found at a very low frequency in human blood, which explains the limited number of studies conducted so far [145]. It has been shown that these cells can influence B cells maturation and induce an increase in IgM production [146].

Most studies do not differentiate between the subgroups, which can limit the understanding of the specific functions of each subpopulation. Studies have shown a positive association between the infiltration of γδ T lymphocytes and favorable clinical outcomes in various subtypes of breast cancer, except for triple negative [147]. In contrast, a significant association has been demonstrated between the higher infiltration of γδ cells in breast cancer tissue and the histological grade of the tumor, suggesting a potential role in characterizing aggressiveness [148]. Additionally, the high infiltration of these cells has been associated with more advanced stages, HER2+ status, and high rates of lymph node metastasis, attributed to the secretion of IL-17, which promotes the proliferation of cancer cells, thus contributing to breast tumor progression [136,149].

### 4.3. B Lymphocytes

B cells play a potentially crucial role in the immune response against cancer by producing specific antibodies targeting tumor cell antigens [31]. However, the clinical significance of assessing B lymphocytes in breast cancer is still not well established due to insufficient robust data. Most studies have struggled to establish a clear link between B cell subtypes and prognosis, making it challenging to draw definitive conclusions on their clinical relevance.

Tumor-infiltrating B cells (TIBs) constitute approximately 20% of all immune-infiltrating cells in breast cancer, a significant proportion compared to normal breast tissue (around 3%) [150,151]. Within the TME, these cells play a multifaceted role, supporting T cell function through antigen presentation and antibody secretion. However, B cell populations are often overlooked in assessments of immune infiltrates in breast cancer [150].

Following antigen activation, B cells differentiate into antibody-secreting plasma cells. IgG, comprising roughly 75% of antibodies in human serum, is predominant among the five human immunoglobulin (Ig) classes [152,153]. In recent years, B cell infiltration in breast cancer patients has been primarily associated with a favorable prognosis [154,155,156].

The anti- and pro-tumor activities of the mentioned cells are summarized in Table 1.

### 4.4. Non-Cellular Components

In addition to the importance of the direct activity of the immune cells, it is worth mentioning the determinant role of their interaction with non-cellular components of breast cancer’s TME. This dynamic interplay influences tumor progression, immune response, and consequently, therapeutic responses [157]. While tumor cells, immune cells, and stromal cells play significant roles, non-cellular components such as the extracellular matrix, cytokines, growth factors, exosomes, metabolites, and the presence of hypoxia, are equally crucial in determining the effectiveness of treatments, including immunotherapies [2].

The extracellular matrix plays a fundamental role in the spatial organization of the TME, providing structural support and acting as an intermediary in cellular interactions and signaling [158]. It is primarily composed of collagen, fibronectin, laminin, elastin, glycoproteins, and proteoglycans, and is produced by stromal cells such as fibroblasts, epithelial cells, and endothelial cells. Additionally, it serves as a reservoir of proteins that regulate cell growth, influencing processes such as proliferation, differentiation, and cell migration [159].

In breast cancer, the dysregulation of the extracellular matrix leads to an increased deposition of components, such as collagen and fibronectin, which increases tissue stiffness. This increase in stiffness facilitates tumor cell migration, promoting invasion into adjacent tissues [160]. Furthermore, extracellular matrix remodeling involves not only collagen accumulation, but also matrix metalloproteinases (MMPs) and lysyl oxidase (LOX), which alter the biochemical and mechanical properties of the TME, fostering an aggressive tumor phenotype [161]. Stiffness measurements show that the invasive front of HER2-positive and triple negative breast cancers is significantly stiffer than that of luminal A and B tumors [159,162].

Extracellular matrix alterations promote tumor progression and contribute to therapy resistance by impairing immune cell function and limiting drug penetration. Targeting extracellular matrix remodeling could improve therapeutic efficacy and clinical outcomes.

Tumor-derived exosomes facilitate communication between tumor cells and the TME by transferring small RNAs, proteins, and lipids, influencing stromal cell behavior, extracellular matrix remodeling, and tumor angiogenesis [157,163]. They also modulate immune responses by expanding immunosuppressive cells, such as Treg cells and MDSCs, suppressing antigen presentation, and promoting immune evasion.

In breast cancer, exosomes contribute to therapy resistance by reducing tumor cell sensitivity to chemotherapy and immunotherapy. They activate STAT3 in bone marrow cells, leading to CXCR4 downregulation and differentiation into MDSCs, which inhibit T cell proliferation [164]. Notably, exosomes from drug-treated tumor cells can transfer resistance to untreated cells within the same microenvironment, reinforcing their role in tumor progression and therapy resistance [165].

### 4.5. Cellular Interactions in the Breast Cancer Microenvironment

**Figure 2:**
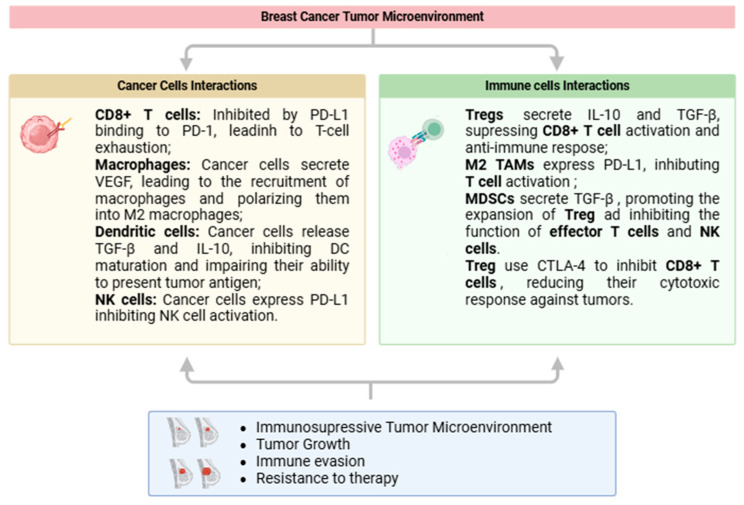
A schematic representation of cellular interactions in the breast cancer tumor microenvironment. Created with BioRender.com.

In the tumor microenvironment of breast cancer, immune cells interact in a complex manner, contributing both to the immune response against the tumor and its progression. The ability of immune cells to recognize and eliminate malignant cells is often compromised by immunosuppressive mechanisms that favor immune evasion. The main cellular interactions that characterize this immunosuppressive environment include CD8+ T cells, NK cells, and M2 macrophages, which together maintain the state of immunosuppression and promote tumor progression (Figure 2). Understanding these interactions is crucial for the development of therapeutic strategies aimed at reversing immunosuppression and restoring the immune system’s ability to fight cancer.

CD8+ T lymphocytes are crucial for the destruction of malignant cells, as they have the ability to recognize and eliminate tumor cells through their cytotoxic activity. However, this function is often compromised in the tumor microenvironment, where various immunosuppressive mechanisms interfere with their activity [31]. The interaction between the PD-L1 protein on tumor cells and the PD-1 receptor on T cells plays a critical role in this process. This interaction induces an exhaustion state in T cells, characterized by a reduced production of essential cytokines, such as IFN-γ and TNF-α, and cytotoxic molecules, like perforins and granzymes, which are responsible for destroying malignant cells [166].

Moreover, TAMs, predominantly of the M2 phenotype, significantly contribute to maintaining this immunosuppressive state. These macrophages secrete immunosuppressive cytokines, such as IL-10 and TGF-β, which not only inhibit the function of CD8+ T cells but also promote a microenvironment that favors tumor progression [30,32].

Th2 cells, traditionally associated with the secretion of IL-4, IL-6, and IL-10, play a role in the tumor microenvironment, as these cytokines activate M2 macrophages. The highly proliferative environment generated by these cells favors the polarization of the TAMs phenotype, further contributing to the suppression of an effective immune response against the tumor [107,108].

NK cells, which play an important role in the elimination of tumor cells without prior antigen recognition, also have their function severely impaired in the breast cancer tumor microenvironment [45]. The increased expression of HLA-G and PD-L1 on tumor cells interferes with the NK cells’ ability to recognize them, preventing their activation and, consequently, their cytotoxic function [167]. In addition, the secretion of immunosuppressive cytokines, such as TGF-β, reduce the expression of the NKG2D receptor, which is essential for activating NK cells, further diminishing their ability to eliminate malignant cells [168].

This complex network of cellular interactions creates a tumor microenvironment that favors immune evasion and cancer progression. The balance between effector cells, such as CD8+ T cells and NK cells, and immunosuppressive cells, such as M2 macrophages, is crucial for the clinical outcome of the disease. A detailed understanding of these interactions has been the key to developing immunotherapies aimed at reversing immunosuppression in the tumor microenvironment and restoring the immune system ability to recognize and eliminate tumor cells. These strategies include therapies that block immune checkpoints, such as PD-1/PD-L1 inhibitors, as well as treatments targeted at modulating the activation of macrophages and NK cells, with the goal of restoring an effective immune response and improving disease control.

### 4.6. Immune Microenvironment Differs in Breast Cancer Subtypes

**Figure 3:**
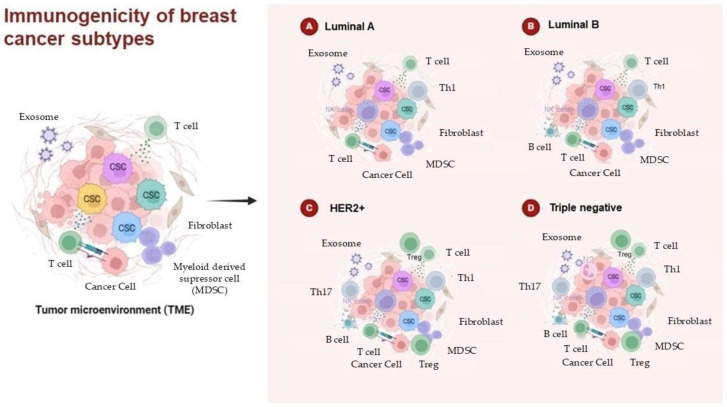
The immunogenicity of breast cancer subtypes. Schematic representation of the immune components within the TME of breast cancer, highlighting the different immune cell populations across different breast cancer subtypes. Created with BioRender.com.

Breast cancer is a highly heterogeneous disease, characterized by diversity in both the molecular properties of tumor cells and the composition of the TME. The TME not only influences tumor biology but also the patient’s response to therapies, such as chemotherapy, immunotherapy, and hormonal therapy. The interaction between tumor cells and immune cells within the TME is a crucial factor in cancer progression, metastasis, and therapeutic responses. Differences in the TME can vary significantly between breast cancer subtypes, and these differences have important clinical implications (Figure 3).

In Luminal A breast cancer, there is a predominance of NK cells, which play a key role in immune surveillance, with a tendency for a higher presence of this cell type compared to Luminal B subtype [56]. Additionally, the presence of CD4+ T cells and Th1 cells is more pronounced in Luminal A, and previous studies indicate a positive correlation between Th1 cells and tumor proliferation, suggesting that Th1-mediated immune responses might influence the proliferative behavior of these tumors [56]. The infiltration of Tc is also more frequent in Luminal A tumors and is often associated with better prognoses [102,133].

On the other hand, Luminal B breast cancer tends to present a more suppressive immune microenvironment, with a higher presence of Treg cells, which may play an important role in immune evasion [122,123]. Although Tregs may be protective in the early stages of carcinogenesis by preventing the transition to invasion, their presence in more advanced tumors can promote immune suppression, favoring tumor growth and metastasis.

The immune microenvironment in HER2+ cancer is distinct, with a significant infiltration of pDCs in the peripheral blood of patients with breast cancer [86]. While the prognostic role of these cells remains unclear, studies suggest they play a role in modulating the immune response and may influence tumor evolution. Furthermore, the presence of Tregs and macrophages in HER2+ tumors is also associated with a more immunosuppressive environment, promoting cancer progression.

Triple negative is one of the most aggressive and challenging subtypes to treat, as it lacks the expression of hormonal receptors and HER2, limiting therapeutic options [17]. The tumor microenvironment in triple negative is characterized by a more intense immune infiltration, with a significant increase in TAMs, which play a crucial role in metastatic progression [34,38]. In addition, the presence of neutrophils, stimulated by cytokines, such as TGF-β, is more prevalent in this subtype and contributes to a tumor environment that favors invasion and metastasis [67].

MDSCs, particularly M-MDSCs, are also increased in triple negative and are associated with a poorer prognosis due to their ability to suppress the anti-tumor immune response and facilitate metastasis [77,78,79]. The presence of these cells may interfere with immune responses, promoting a tumor-friendly environment.

Moreover, triple negative is characterized by elevated IL-17 expression, a cytokine produced by Tc that is associated with tumor cell proliferation, contributing to tumor progression and lymph node metastasis [136,149].

The differences in the immune microenvironment across breast cancer subtypes have a direct impact on therapeutic response. Subtypes with high infiltration of Tregs and TAMs, such as triple negative and HER2+, may respond less favorably to immune-activating therapies, such as immune checkpoint inhibitors (e.g., anti-PD-1/PD-L1) [122,123]. In contrast, subtypes like Luminal A, with a higher presence of NK cells and Tc cells, may respond better to treatments that aim to activate the immune system due to their less immunosuppressive microenvironment.

In summary, the variations in the immune microenvironment across breast cancer subtypes play a crucial role in tumor progression, metastasis, and therapy responses. These differences should be taken into consideration when developing more targeted and effective therapies, with the goal of personalizing treatment for each breast cancer patient based on their tumor’s immune profile.

## 5. Immune Checkpoint Molecules

The immune system is meticulously regulated by immune checkpoints, which protect healthy cells from immune system-mediated destruction [169]. Cytotoxic T-lymphocyte-associated protein 4 (CTLA-4), also known as CD152, is a protein belonging to the immunoglobulin superfamily, found exclusively in T cells. Structurally akin to CD28, a co-stimulatory receptor of T cells, CTLA-4 is an immunosuppressive molecule that governs the T cells functionality, curbing damage caused by immune cells and averting autoimmune reactions. An elevated CTLA-4 expression in breast cancer cells correlates with a poorer prognosis [170]. Studies indicate a heightened CTLA-4 expression in invasive ductal breast cancer compared to in situ carcinomas, signifying escalating immunological suppression during tumor progression [171].

Programmed cell death protein 1 (PD-1), or CD279, is a member of the CD28 family renowned for its role in homeostatic processes, delivering inhibitory cues upon engaging with its ligands (PD-L1 and PD-L2). PD-1 plays a pivotal role in programmed cell death signaling. Like CTLA-4, it is believed to serve as a negative regulator of T cell function. PD-1 expression has been observed in various immune cells within the TME, including monocytes, DCs, NK cells, T cells, and B cells [172,173]. It has been reported that PD-L1 expression is associated with clinicopathological parameters of a high risk and poor prognosis [174].

Recently, CD39 has been identified as a critical target of the immune system. CD39 converts extracellular adenosine triphosphate (ATP) into adenosine [175,176]. Adenosine suppresses T cell immune activity by inhibiting effector responses and inducing the expansion of immunosuppressive cells [78,177]. Thus, an increased expression of tumor-derived CD39 correlates with a poor prognosis in various cancer types [178,179,180]. In breast cancer, CD39 expression has been demonstrated to correlate with a worse clinical prognosis [62].

CD73, also known as ecto-5′-nucleotidase, is a crucial enzyme that converts extracellular ATP to adenosine, thus limiting the degradation of ATP [181,182]. CD73 is overexpressed in the TME of several cancers, including breast cancer. Moreover, growing evidence suggests that the CD73-adenosine pathway is vigorously involved in breast cancer progression. Choi et al. [183] demonstrated that CD73 is associated with tumor progression, positive PgR status, and nodal metastasis. However, in breast cancer, the prognostic significance of CD73 remains controversial.

In addition to enhancing the metastatic capabilities of tumor cells, the CD39/CD73 axis creates an immunosuppressive effect on macrophages, neutrophils, and T cells [184]. Tumor cells have the capacity to express these inhibitory molecules, thereby fostering an immunosuppressive TME that impedes effective anti-tumor immune responses [185,186]. In breast cancer, the expression patterns of immune checkpoint molecules emerge as crucial focal points in the exploration of novel therapeutic avenues [186].

## 6. Current Treatment Regimens in Breast Cancer: Subtypes and Limitations

**Figure 4:**
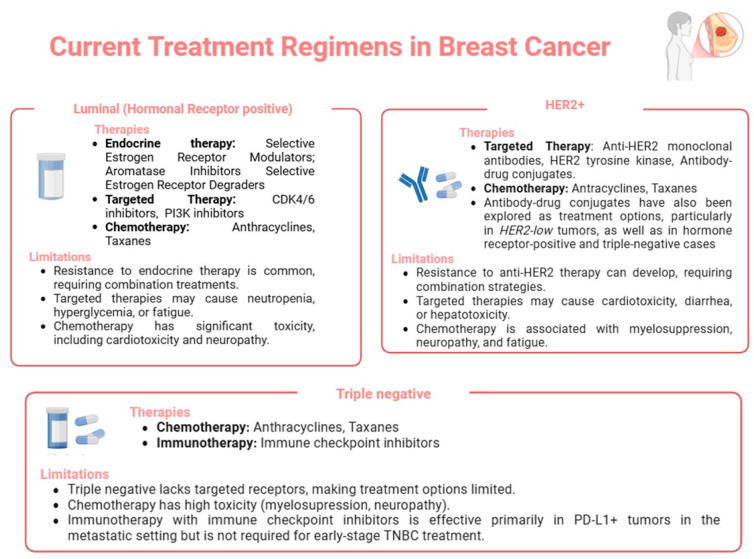
Current treatment regimens for different breast cancer subtypes and their limitations. The figure summarizes the main therapeutic approaches for Luminal (hormone receptor-positive), HER2+, and triple negative breast cancer, highlighting chemotherapy, targeted therapy, endocrine therapy, and immunotherapy where applicable. The associated limitations of each approach, such as resistance mechanisms and treatment-related toxicities, are also outlined. Created by BioRender.com.

Breast cancer treatment has evolved significantly in recent years, with the molecular classification of breast cancer into subtypes enabling the development of more targeted and effective therapies (Figure 4). Despite these advances, several limitations persist, including treatment resistance, adverse effects, and a lack of effective options for certain aggressive subtypes, such as triple negative breast cancer [187]. The personalization of treatment, the use of biomarkers, and combination therapies have shown promise in overcoming these limitations. However, significant challenges remain, particularly in preventing resistance and disease recurrence.

Luminal breast cancer, characterized by hormone receptor positivity, is primarily treated through endocrine therapy, which targets the hormonal axis. Selective estrogen receptor modulators, like tamoxifen, and aromatase inhibitors, such as letrozole and exemestane, have proven effective in reducing tumor proliferation by blocking estrogen receptor activation [188]. In cases of resistance to endocrine therapy, CDK4/6 inhibitors, such as palbociclib, have been integrated into treatment strategies to prevent cell cycle progression [187,189]. However, acquired resistance remains a major challenge, driven by mechanisms, such as ER mutations, the activation of compensatory signaling pathways (e.g., HER2 or PI3K/Akt), and the altered expression of transcriptional co-regulators [188,190]. Additionally, adverse effects, including bone density loss and joint disorders, can negatively impact patients’ quality of life and adherence to therapy [188]. Current research is focused on identifying novel therapeutic strategies to bypass resistance mechanisms while minimizing side effects.

HER2+ breast cancer is driven by an overexpression of the HER2 tyrosine kinase receptor, which activates signaling pathways that promote cell proliferation and survival. Targeted therapies, such as the monoclonal antibodies trastuzumab and pertuzumab, effectively inhibit HER2 activation, while antibody–drug conjugates, like trastuzumab emtansine (T-DM1), enhance efficacy by combining the HER2 blockade with chemotherapy [191]. Additionally, tyrosine kinase inhibitors (TKIs), such as lapatinib, can be used in trastuzumab-resistant cases. However, resistance to HER2-targeted therapy remains a significant limitation, with mechanisms including HER2 mutations, the activation of alternative signaling pathways (e.g., PI3K/Akt or MAPK), and the altered expression of proteins that counteract antibody activity [192]. Moreover, cardiotoxicity associated with prolonged trastuzumab use necessitates careful cardiac monitoring, limiting its applicability in some patients [193]. Ongoing research focuses on developing more effective, less toxic therapies with a reduced resistance potential.

Triple negative breast cancer presents major therapeutic challenges due to the absence of molecular targets, such as ER, PR, and HER2, which limit treatment options. Chemotherapy remains the mainstay of treatment, with agents, such as paclitaxel and doxorubicin, commonly used to control tumor growth [194]. However, triple negative is highly aggressive, associated with early metastasis, and has a poorer prognosis than other subtypes. Resistance to chemotherapy may arise through mechanisms, such as DNA repair pathway activation (e.g., homologous recombination) or the overexpression of anti-apoptotic proteins, such as BCL-2 [195]. More recently, immunotherapy has demonstrated promising results, particularly with immune checkpoint inhibitors, such as pembrolizumab (a PD-1 inhibitor), which enhances immune responses against tumors [196]. Combination approaches, namely chemotherapy with immunotherapy or targeted therapies like PARP inhibitors, for BRCA-mutated tumors, represent an emerging research area. Although these combinations have improved response rates, challenges related to resistance and toxicity persist [195].

Breast cancer treatment is highly dependent on molecular subtype and, while available therapies have significantly improved patient outcomes, challenges such as treatment resistance and adverse effects remain major obstacles. Advancements in precision medicine, biomarker-driven therapies, and emerging combination strategies, including immunotherapy and gene therapies, hold great promise. Overcoming these challenges will require a continuous and multidisciplinary effort, but the future of breast cancer treatment appears increasingly promising.

## 7. The Potential of New Therapeutic Approaches Targeting the Immune System

Immunotherapy encompasses a range of strategies to harness the immune system to eliminate tumor cells. However, its application in solid tumors still faces significant challenges due to the tumor’s ability to evade immune surveillance, creating immunosuppressive environments [21]. Immunotherapy comprises a wide range of therapeutic strategies, including immune checkpoint inhibitors, chimeric antigen receptor (CAR)-T cell therapy, and antibody-based therapy [197]. Due to tumor heterogeneity, the response to immunotherapy is diverse. Recent research, focused on each breast cancer subtype, has confirmed the need for distinct therapeutic approaches [198].

Immune checkpoint inhibitors work by blocking immune suppressor molecules like PD-1, PD-L1, and CTLA-4. In fact, antibodies blocking the PD-1/PD-L1 interaction can inhibit tumor growth. Recently, the FDA has approved anti-PD-L1 (atezolizumab, avelumab, and durvalumab) and anti-PD-1 (nivolumab, pembrolizumab, cemiplimab, and dostarlimab-gxly) medications for the treatment of breast cancer [199]. Avelumab, for example, besides blocking the PD-L1/PD-1 interaction, reducing the immunosuppressive state of the TME and restoring T cell activity, also mediates antibody-dependent cell-mediated cytotoxicity [200]. Additionally, pembrolizumab has been approved for the treatment of triple negative breast cancer in combination with chemotherapy in early-stage breast cancer [201].

Clinical trials regarding immune checkpoint inhibitors and monoclonal antibodies, including in the neoadjuvant context, are still ongoing, as detailed in Table 2.

## 8. Future Research Directions

Breast cancer treatment is highly dependent on the molecular subtype, and while available therapies have significantly improved patient outcomes, challenges such as treatment resistance and adverse effects remain major obstacles. Advancements in precision medicine, biomarker-driven therapies, and emerging combination strategies, including immunotherapy and gene therapies, hold great promise. Overcoming these challenges will require a continuous and multidisciplinary effort, but the future of breast cancer treatment appears increasingly promising.

As our understanding of breast cancer and its interaction with the tumor microenvironment continues to evolve, several promising areas of research are emerging that could help improve current treatment strategies and address existing limitations. While significant progress has been made, much remains to be explored regarding the role of immune cells in tumor progression and treatment resistance. The tumor microenvironment has proven to be a critical determinant in tumor progression, immune evasion, and therapy resistance. However, the precise role of various immune cells, such as tumor-associated macrophages, regulatory T cells, and dendritic cells, remains poorly understood in many breast cancer subtypes. Future research aimed at unraveling how these cells contribute to tumor progression and immune suppression could provide valuable insights for the development of immunotherapies.

Although immunotherapy has shown promising results in the treatment of certain breast cancer subtypes, such as triple negative breast cancer, its efficacy in hormone receptor-positive subtypes, such as hormone receptor+/HER2−, remains limited. Future studies should focus on how immunotherapies can be combined with other targeted therapies, such as HER2 inhibitors or CDK4/6 inhibitors, to enhance their effectiveness. Additionally, exploring the role of immune checkpoint inhibitors in overcoming resistance mechanisms across various breast cancer subtypes could lead to the development of more effective treatment regimens.

The tumor microenvironment is not only a barrier to effective therapies but also a potential therapeutic target. Research on how the tumor microenvironment influences treatment resistance, particularly in relation to hypoxia, extracellular matrix remodeling, and metabolic reprogramming, could lead to new therapeutic strategies. Targeting stromal components and enhancing immune cell recruitment to the tumor microenvironment may offer new opportunities to improve therapeutic outcomes in breast cancer.

With the increasing availability of genomic and molecular profiling, there is a growing need for personalized therapeutic approaches in breast cancer. Future studies should focus on identifying biomarkers that predict responses to specific treatments, including chemotherapy, targeted therapies, and immunotherapies. Developing predictive models for treatment plans tailored to each patient could significantly improve outcomes and reduce unnecessary side effects.

Another major challenge in breast cancer treatment is the development of resistance to therapies, especially in metastatic disease. Exploring novel drug delivery systems, such as nanoparticle-based platforms, could help overcome issues related to drug resistance and poor drug bioavailability. Additionally, more studies are needed to understand the molecular mechanisms underlying drug resistance and to identify strategies to overcome it, such as using combination therapies or novel agents targeting resistant tumor cell populations.

While many promising results have emerged in pre-clinical models, robust clinical trials are still required to validate these approaches. Future research should focus on the design and implementation of clinical trials that explore new combination therapies, innovative immunomodulators, and the potential of personalized medicine in the context of breast cancer. Translating pre-clinical findings into clinical applications remains a critical step toward improving patient outcomes. By addressing these key issues, future research has the potential to significantly enhance breast cancer treatment, offering more effective and personalized therapeutic strategies that could lead to better patient outcomes and quality of life.

## 9. Conclusions

This review has gathered the current knowledge on the intricate interactions between immune cells and the tumor microenvironment in breast cancer, emphasizing their significant influence on the disease’s development, progression, and prognosis. Immune cells, such as neutrophils, lymphocytes, and dendritic cells, play diverse roles in both promoting and inhibiting tumor growth. However, due to the high heterogeneity of breast cancer, the specific roles of these cells remain incompletely understood. Understanding these interactions offers promising potential for the development of new therapeutic strategies, including immune checkpoint inhibitors. Future research should prioritize elucidating the specific mechanisms that can be targeted for therapeutic interventions, aiming to create a tumor microenvironment that supports the elimination of malignant cells and prevents tumor progression.

## Figures and Tables

**Table 1:** Anti and pro-tumor activity of the immune cells in cancer.

Immune Cell Type	Anti-Tumor Activity	Pro-Tumor Activity	Breast Cancer Subtypes	Reference
Macrophages	**M1 macrophages**: Inhibit invasion and proliferation.	**M2 macrophages**: Produce anti-inflammatory cytokines (IL-10 and TGF-β); induce Treg.	M2 macrophages are predominant in triple negative.	[24,25,26]
Natural killer cells	Recognize and destroy tumor cells through cytotoxic mechanism.	Expression of VEGF and MMP-9, promoting angiogenesis.	Predominant in Luminal A.	[50,51,55]
Neutrophils	**N1 neutrophils**: Exhibit enhanced phagocytic capacity; produce pro-inflammatory cytokines (TNF-α, CCL13).	**N2 neutrophils**: Reduced cytotoxicity; secrete high levels of pro-tumoral factors (VEGF, IL-18, and MMP-9).	Predominant in triple negative.	[63,64]
MDSC	-	Suppression of T cells and NK cells; induction of oxidative stress; secretion of ROS, VEGF, and MMPs.	MDSCs accumulate in the TME of triple negative breast cancer.	[70,71]
Dendritic cells	**cDCs**: Potent antigen-presenting cells. **pDCs**: Produce type I interferon; activation of other immune cells.	**pDCs**: Expression of immunosuppressive molecules (PD-L1, ICOS); promotion of Treg expansion.	Predominant in HER2+.	[82,83,84,85]
Innate lymphoid cells	**ILC1**: Expression of IFN-γ and TNF-α. **ILC3**: Secrete IL-22.	**ILC2**: Produce effector cytokines (IL-5, IL-9, IL-13); induce polarization to M2 macrophages. **ILC3**: Secrete IL-17.	-	[89,90,91]
T cells	**Th1**: Produce IFN- γ; activate M1 macrophages. **Tc**: cytotoxin release (perforin and granzyme B); produce pro-inflammatory cytokines (IFN-γ and TNF-α). **Vδ2**: Cytotoxic functions against tumor cells; attenuate angiogenic signaling processes.	**Th2**: Secrete IL-4, IL-6, and IL-10; activate M2 macrophages. **Th17**: Secrete pro-inflammatory cytokines (IL-17); stimulate inflammatory mediators like MMPs. **Treg**: Secretion of immunosuppressive cytokines (IL-10 and TGF-β). **Vδ1**: Suppress T cells.	Tregs are predominant in HER2+ and triple negative.	[78,104,106,107,108,110,111,129,136]
B cells	Produce specific antibodies targeting tumor cell antigens.	-	-	[31]

**Table 2:** Ongoing clinical trials on immunotherapeutic immune checkpoint inhibitors in breast cancer.

Therapy	Target	Tumor Type	Setting	Estimated Study Completion Date	Phase	NCT Number	Reference
Nivolumab + Cabozantinib	PD-1	Triple negative	Metastatic	2025	II	NCT 033414684	[202]
Transtuzumab	Anti-HER2	HER2+	Residual tumor in the breast or axillary lymph nodes	2025	III	NCT 01772472	[203]
Pembrolizumab + Chemotherapy	PD-1	Triple negative	Untreated early stage	2025	III	NCT 03036488	[204]
Durvalumab + Tremelumumab	PD-1	HER2−	Stage II–III	2024	I	NCT 03132467	[205]
Atezolizumab + capecitabine	PD-1	Triple negative	Residual tumor after neoadjuvant chemotherapy	2027	II	NCT 03756298	[206]
Transtuzumab emtansinabe	Anti-HER2	HER2+	Unresectable or metastatic	2025	III	NCT 03529110	[207]
Paclitaxel + Carboplatin + Durvalumab	PD-1	Triple negative	Previously untreated locally recurrent inoperable or metastatic	2025	I/II	NCT 03616886	[208]

## Abbreviations

CCL	CC motif chemokine ligand
cDC	Conventional dendritic cells
CSF	Colony-stimulating factor
CTLA-4	Cytotoxic T-Lymphocyte-Associated Protein 4
ER	Estrogen Receptor
FoxP3	Forkhead box P3
HER2	Human Epidermal Growth Factor Receptor 2
HR	Hormone Receptor
IFN	Interferon
Ig	Immunoglobulin
IL	Interleukin
ILC	Innate Lymphoid cells
KIR	Killer immunoglobulin-like receptor
M1	Macrophages M1
M2	Macrophages M2
MDSCs	Myeloid-Derived Suppressor Cells
Mha	Hemophagocytic macrophages
M-MDSCs	Monocytic myeloid-derived suppressor cells
Mox	Oxidative macrophages
Mreg	Regulatory macrophages
N1	Neutrophils N1
N2	Neutrophils N2
NCRs	Natural cytotoxicity receptors
pDC	Plasmacytoid dendritic cells
PgR	Progesterone Receptor
PMN-MDSC	Polymorphonuclear myeloid-derived suppressor cells
PD-L	Programmed Death ligand
ROS	Reactive oxygen species
TAMs	Tumor-associated macrophages
TCR	T cell receptor
Tfh	Follicular regulatory T cells
Tfr	Follicular regulatory T cells
Th	Helper T cells
Tc	Cytotoxic T cells
TGF-β	Transforming growth factor beta
TME	Tumor Microenvironment
TNF-α	Tumor necrosis factor alpha
VEGF	Vascular endothelial growth factor
